# Adenoviruses in Fecal Samples from Asymptomatic Rhesus Macaques, United States

**DOI:** 10.3201/eid1807.111665

**Published:** 2012-07

**Authors:** Soumitra Roy, Arbansjit Sandhu, Angelica Medina, David S. Clawson, James M. Wilson

**Affiliations:** University of Pennsylvania, Philadelphia, Pennsylvania, USA

**Keywords:** adenovirus, rhesus, macaques, fiber, hexon, DNA, polymerase, United States, feces, stool, fecal samples, viruses, asymptomatic, subclinical infection, PCR, monkeys

## Abstract

Adenoviruses can cause infectious diarrheal disease or respiratory infections in humans; 2 recent reports have indicated probable human infection with simian adenoviruses (SAdVs). To assess the possibility of animal-to-human transmission of SAdVs, we tested fecal samples from asymptomatic rhesus macaques housed in 5 primate facilities in the United States and cultured 23 SAdV isolates. Of these, 9 were purified and completely sequenced; 3 SAdV samples from the American Type Culture Collection (SAdV-6, SAdV-18, and SAdV-20) were also completely sequenced. The sequence of SAdV-18 was closely related to that of human adenovirus F across the whole genome, and the new isolates were found to harbor 2 fiber genes similar to those of human adenovirus (HAdV) strains HAdV-40 and HAdV-41, which can cause infectious diarrhea. The high prevalence of adenoviruses in fecal samples from asymptomatic rhesus macaques and the similarity of the isolates to human strains indicates the possibility of animal-to-human transmission of SAdVs.

## Abstract 2

Isolates contained fiber genes similar to those of adenovirus strains that cause infectious diarrhea in humans.

Adenoviruses, which can cause infectious diarrhea or respiratory infections in humans, were isolated from monkeys soon after their initial characterization by Rowe et al. (*1*). Hull et al. (*2**,**3*) identified adenoviruses as one of several classes of viruses found as outgrowths from primary cultures of monkey kidney cells that were being cultivated for propagation of poliovirus. Adenoviruses were also found in rectal swab and fecal samples taken from monkeys in captivity (*4**,**5*).

The classification of monkey adenoviruses was initially done in a manner analogous to that used for human isolates, i.e., on the basis of differences in their ability to agglutinate erythrocytes from different species. Rapoza classified monkey adenoviruses into 4 hemagglutination subgroups on the basis of their differential agglutination properties with respect to their ability to agglutinate rat, rhesus, and guinea pig erythrocytes (*6*). Most viruses tested at the time were found to belong to groups 2 and 3; groups 1 and 4 contained only 1 member each. The current classification of macaque adenoviruses by the International Committee on Taxonomy of Viruses has 1 defined species, simian adenovirus A (SAdV-A), to which SAdV-3 has been assigned. SAdV-1 and SAdV-7, while not considered human viruses, are now classified as human adenovirus G (HAdV-G) because of their similarity to a virus (HAdV-52) isolated from a human patient with gastroenteritis (*7*).

Recently, transmission of a monkey adenovirus from captive titi monkeys to a researcher was reported at the California National Primate Research Center (Davis, CA, USA); the researcher may, in turn, have transmitted it to a family member (*8*). Further, HAdV-52 (*7*) isolated from a patient with gastroenteritis has been found to be very dissimilar to all previously isolated human adenoviruses but closely related to monkey adenoviruses SAdV-1 and SAdV-7. This similarity raises the possibility that this patient was, in fact, infected with a monkey adenovirus.

We have previously found that adenovirus DNA is readily detected in fecal samples from monkeys who have no symptoms of any clinical adenoviral disease (*9*). Given the close physical proximity and intermingling of human and monkey populations in several locales in Asia, Africa, and Latin America, better characterization of monkey adenoviruses is of public health importance. To clarify the nature of adenoviruses shed in the feces of asymptomatic monkeys, we attempted to culture adenoviruses from fecal samples from rhesus macaques (*Macaca mulatta*) housed in 5 primate facilities in the United States.

## Materials and Methods

### Isolation of Novel Adenoviruses from Rhesus Macaques

Approximately 350 rhesus macaque fecal samples were processed: 150 from Covance (Vienna, VA, USA), 100 from the New England Primate Research Center (Southborough, MA, USA), and 98 from the Tulane National Primate Research Center (Covington, LA, USA). Additional samples were obtained from rhesus macaques housed at the Oregon National Primate Research Center (Beaverton, OR, USA) and the primate facility of the Gene Therapy Program at the University of Pennsylvania (Philadelphia, PA, USA).

Samples were suspended in Hanks’ Balanced Salt Solution (Life Technologies, Grand Island, NY, USA), particulates were removed by centrifugation, and the supernatants were sterile filtered through 0.2-µm syringe filters. We injected 100 µL of each filtered sample into BS-C-1 and LLC-MK2 cells grown in culture medium containing 1% Penn-Strep (Mediatech, Herndon, VA, USA) and 50 µg/mL gentamicin. After ≈1–2 weeks, if cytopathic effects typical of adenovirus infection were seen, the presence of adenoviruses in the cultures was confirmed by PCR amplification of an internal 1.9 kb of the hexon, including the region encompassing the hypervariable regions, which is predominantly responsible for conferring serotype specificity. The primer pair used for PCR was 5′-CAGGATGCTTCGGAGTACCTGAG-3′ and 5′-TTGGCNGGDATDGGGTAVAGCATGTT-3′. The sequence obtained from this region was used to make an initial determination of adenoviral species and novelty of the serotype. Adenovirus isolates were propagated to high titer and purified on cesium chloride gradients by using standard procedures. Viral DNA obtained from purified virus preparations was completely sequenced (QIAGEN, Hilden, Germany).

### Adenoviruses and Cell Culture from ATCC

We obtained 3 adenoviruses from the American Type Culture Collection (ATCC). SAdV-6 (catalog number VR-353, originally deposited as SV-39) had been isolated from *Macaca* spp. monkeys (*6**,**10*). SAdV-18 (catalog number VR-943) (*4**,**11*) and SAdV-20 (catalog number VR-541, originally deposited as simian adenovirus V340) had been isolated from vervet monkeys (*Cercopithecus aethiops*) (*12*). BS-C-1 (*C. aethiops* monkey kidney cells, ATCC catalog number CCL-26) or LLC-MK2 (*M. mulatta* monkey kidney cells, ATCC catalog CCL-7) were purchased from ATCC and maintained in culture as recommended.

### Sequence Analysis

Propagation and purification were performed for 23 isolates as shown in Table 1; 9 of the isolates were completely sequenced. In addition, we propagated, purified, and sequenced the 3 monkey adenoviruses (SAdV-6, SAdV-18, and SAdV-20) from ATCC. All analyses were conducted at the www.phylogeny.fr sequence analysis server. Nucleotide sequence alignments for each open reading frame (ORF) (after excising introns if necessary) were completed by using ClustalW version 2.0.3, and the alignments were refined by using Gblocks version 0.91b. The alignments were used to construct phylogenetic trees using PhyML version 3.0 aLRT, under the HKY85 model. The resulting trees were rendered by using Treedyn 198.3. Protein sequence alignments and phylogenetic trees were constructed by using the Vector NTI (Life Technologies) and CLC Bio (CLC Bio, Aarhus, Denmark) software packages.

**Table 1:** Characterization of adenovirus isolates from rhesus fecal samples obtained from primate facilities in the United States*

Isolate	Source†	Particle titer	Total yield	Sequence analysis
A1123	ONPRC	4.0 × 10^11^	9.6 × 10^11^	Hexon similar to SAdV-3
A1128	Covance	6.0 × 10^11^	1.3 × 10^12^	Hexon similar to SAdV-3
A1129	Covance	6.1 × 10^11^	3.8 × 10^13^	Hexon (A1128)
**A1139**	Covance	4.7 × 10^12^	1.8 × 10^12^	Full genome
A1161	Covance	2.0 × 10^12^	4.3 × 10^13^	Hexon similar to SAdV-48
**A1163**	Covance	5.4 × 10^12^	5.4 × 10^12^	Full genome
A1166	Covance	2.8 × 10^12^	1.3 × 10^11^	Hexon (A1161)
A1169	Covance	1.1 × 10^11^	2.5 × 10^13^	Hexon (A1161)
**A1173**	Covance	5.2 × 10^12^	2.3 × 10^12^	Full genome
A1179	Covance	4.6 × 10^12^	1.0 × 10^13^	Hexon (A1128)
**A1258**	GTP	1.8 × 10^12^	2.6 × 10^12^	Full genome
A1261	GTP	8.7 × 10^11^	9.0 × 10^12^	Hexon (A1285)
**A1285**	GTP	2.7 × 10^12^	2.0 × 10^13^	Full genome
**A1296**	Covance	5.4 × 10^12^	7.3 × 10^11^	Full genome
A1297	Covance	3.7 × 10^11^	7.0 × 10^11^	Hexon (A1163)
**A1312**	Covance	6.1 × 10^11^	8.4 × 10^11^	Full genome
A1313	TNPRC	6.5 × 10^11^	1.4 × 10^13^	Hexon similar to A1312
**A1327**	TNPRC	2.4 × 10^12^	1.0 × 10^12^	Full genome
A1328	NEPRC	6.7 × 10^11^	2.7 × 10^12^	Hexon similar to SAdV-49
A1329	TNPRC	3.7 × 10^12^	9.2 × 10^12^	Hexon (A1312)
**A1335**	NEPRC	6.0 × 10^12^	2.8 × 10^13^	Full genome
A1339	TNPRC	3.1 × 10^12^	5.7 × 10^12^	Hexon (A1163)
A1340	Covance	5.1 × 10^12^	3.1 × 10^13^	Hexon (A1335)

*The total yield and the particle titer obtained from a 50-plate (150-mm) preparation are shown. In each case, the hexon region was PCR amplified and sequenced. **Boldface** indicates the 9 isolates that were completely sequenced. For the other isolates, the hexon sequences that are identical or very closely related (<5% nucleotide sequence dissimilarity) are indicated in parentheses. SAdV, simian adenovirus. † ONPRC, Oregon National Primate Research Center (Beaverton, OR, USA); Covance, Covance, (Vienna, VA, USA); GTP, primate facility of the Gene Therapy Program at the University of Pennsylvania (Philadelphia, PA, USA); TNPRC, Tulane National Primate Research Center (Covington, LA, USA); NEPRC, New England Primate Research Center (Southborough, MA, USA).

## Results

All the macaque adenoviruses that we sequenced were similar to previously sequenced primate adenoviruses with respect to the identity and order of identifiable ORFs organized into defined early and late transcription regions (*13*). The notable differences observed were in structures of the E3 region genes and of the fiber genes.

**Figure 1:**
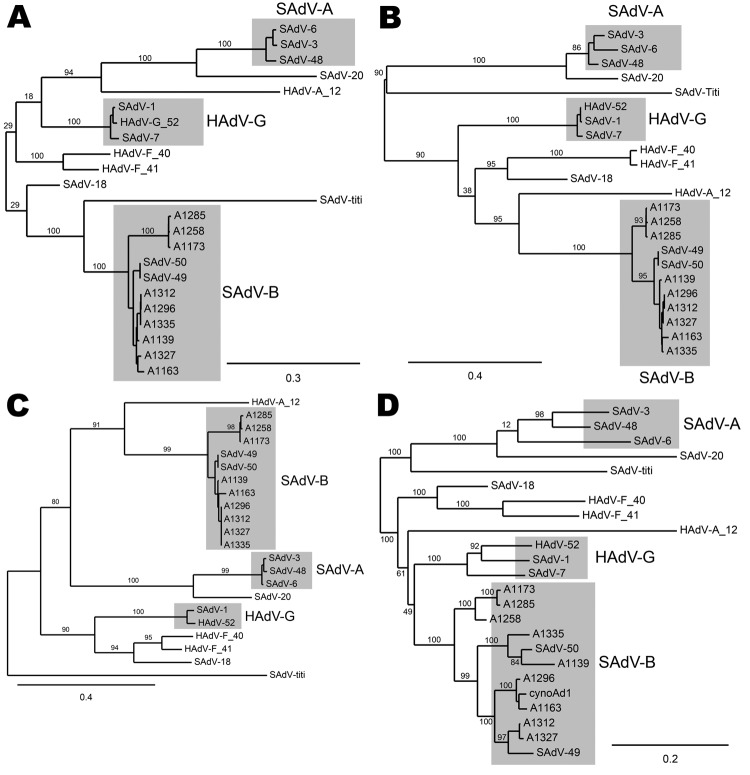
Phylogenetic trees of the genes coding for A) DNA polymerase, B) E4 34K, C) E1a, and D) hexons of macaque adenoviruses identified in study of prevalence of adenoviruses in fecal samples from rhesus macaques, United States. Members of the human adenovirus (HAdV) species HAdV-A (HAdV-12), HAdV-G, and HAdV-F that are thought to have the closest phylogenetic proximity to macaque adenoviruses are included for comparison. Branch support values are indicated. Simian adenoviruses (SAdV) SAdV-1 and SAdV-7 have been grouped together with HAdV-52 into HAdV-G; the other macaque adenoviruses (except for SAdV-18, SAdV-20, and the titi monkey adenovirus) have been grouped into SAdV-A and SAdV-B. SAdV-18 is seen to be closely related to HAdV-F (HAdV-40 and HAdV-41) in all the trees. Scale bars indicate number of substitutions per site.

Phylogenetic analyses of the nucleotide sequences that encode genes of several of the adenoviral proteins showed the sequences were generally concordant with one another. As examples, the phylogenetic trees for the sequences encoding E1a, DNA polymerase, hexon, and E4 34K are shown in Figure 1. The sequences have been compared with each other and with previously sequenced macaque adenoviruses SAdV-1, SAdV-3, SAdV-7, SAdV-48, SAdV-49, SAdV-50, titi monkey adenovirus, and cynomolgus adenovirus 1 (*8**,**9**,**14**–**17*). The human isolate HAdV-52 (*7*), which is known to be closely related to SAdV-1 and SAdV-7, was also included in the analyses. Macaque adenoviruses form a distinct clade when compared with human or ape adenoviruses (*9*). However, HADV-F (HAdV-40 and HAdV-41) and HAdV-A (HAdV-12) are also included in the analyses because these species are the most closely related to macaque adenoviruses (*14**,**15*).

The phylogenetic trees (Figure 1) show that SAdV-6, obtained from ATCC and sequenced, is similar to SAdV-48, which we had previously isolated from stool samples of an apparently healthy macaque (*9*), and to SAdV-3 (*14*). These adenoviruses have been grouped together with SAdV-3 as SAdV-A. The 9 adenoviruses that we isolated from rhesus stools are closely related to one another and to 2 other macaque adenoviruses that we had previously isolated and sequenced, SAdV-49 and SAdV-50 (*9*); these isolates have been grouped as SAdV-B. The adenovirus isolated from a cynomolgus macaque for which a partial sequence (including the hexon) was reported (*16*) also belongs to this group. The adenovirus of unknown origin that recently caused an outbreak of pneumonia and hepatitis in a colony of new world titi monkeys and sickened an animal handler (*8*) is distant from these adenoviruses. SAdV-18, which had been deposited with ATCC as an isolate from a vervet monkey in the 1960s (*11*), was found to be most closely related to HAdV-F members HAdV-40 and HAdV-41 in the protein phylogenies and also for most of the length of its genome (data not shown). HAdV-F is the only human adenovirus species that harbors 2 fiber genes, a feature common in macaque adenoviruses, including HAdV-G members; however, SAdV-18 differs from HAdV-F members in possessing a single fiber gene.

All sequences have been deposited in GenBank. The accession numbers are as follows: simian adenovirus strain A1139, JN880448; strain A1163, JN880449; strain A1173, JN880450; strain A1258, JN880451; strain A1285, JN880452; strain A1296, JN880453; strain A1312, JN880454; strain A1327, JN880455; strain A1335, JN880456; SAdV-6, JQ776547; SAdV-18, FJ025931; and SAdV-20, HQ605912. The accession numbers for other adenovirus sequences used in the analyses are as follows: SAdV-1, NC_006879; SAdV-3, NC_006144; SAdV-7, DQ792570; SAdV-48, HQ241818; SAdV-49, HQ241819; SAdV-50, HQ241820; SAdV-titi, HQ913600; HAdV-2, NC_001405; HAdV-3, NC_011203; HAdV-4, NC_003266; HAdV-12, NC_001460; HAdV-17, HQ910407; HAdV-18, GU191019; HAdV-40, NC_001454; HAdV-41, DQ315364; and HAdV-52, DQ923122.

### Analysis of the E3 Regions

**Figure 2:**
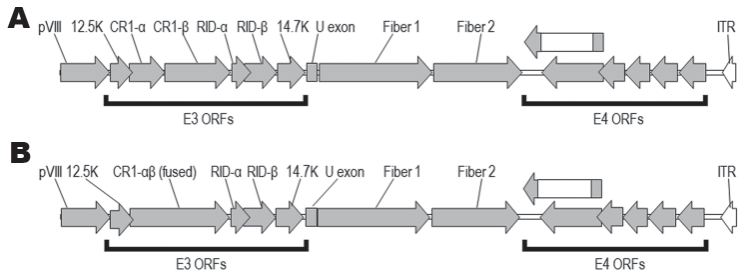
Open reading frames (ORFs) in the right ends of the genomes of 2 macaque adenovirus isolates identified in study of prevalence of adenoviruses in fecal samples from rhesus macaques, United States. A) Isolate A1312 right end (9,606 bp); B) isolate A1139 right end (9,591 bp). ORFs for the E3 proteins CR1-α and CR1-β are present in A1312 but have been combined into a single ORF in A1139. The 2 fiber genes, in which a long fiber protein (fiber 1) is followed by a shorter fiber protein (fiber 2), are also shown. ITR, inverted terminal repeat.

**Figure 3:**
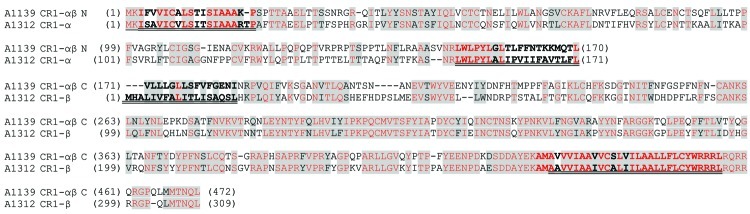
Sequence alignments of a subset of simian adenovirus type B (SAdV-B) isolates identified in study of prevalence of adenoviruses in fecal samples from rhesus macaques, United States. The putative fused E3 CR1-αβ protein from isolate A1139 (see Table 2) and the corresponding separately encoded CR1-α and CR1-β proteins from isolate A1312 are shown. The N-terminal (N) and C-terminal (C) sections of the fused A1139 CR1-αβ proteins have been separately aligned with the A1312 CR1-α and CR1-β proteins, respectively. Gray shading indicates homologous regions, red font indicates identical residues, and underlining indicates hydrophobic regions.

The gene content and disposition (as discerned by the presence of ORFs) of the E3 regions of the 9 adenoviruses isolated from macaques are shown in Table 2. The 12.5K protein of unknown function, as well as the anti-apoptotic RID-α and RID-β, and the 14.7K proteins (*18*), are present in all the newly isolated viruses. They all also harbor homologs of the CR1 proteins that contain conserved domains of unknown function designated CR1 and CR2 (*19*). However we found that in 6 of the newly sequenced adenoviruses, the 2 CR1 proteins (CR1-α and CR1-β) were fused into a single ORF (Figure 2). An example of the fusion is illustrated in Figure 3, in which the fused CR1 protein (designated CR1-αβ) of the adenovirus isolate A1139 E3 region has been aligned with the CR1-α and CR1-β proteins of the E3 region of A1312. The E3 CR1 proteins possess a single putative transmembrane domain near their C-termini and are likely to have arisen by gene duplication (*20**,**21*). The putative transmembrane domain of the CR1-α protein appears to have fused to the hydrophobic (putative) secretion signal of the CR1-β protein. This fused version indicates that the CR1-α and CR1-β proteins are likely disposed on opposite sides of the membrane. One possible model for A1163 CR1-αβ fused protein (based on a prediction by TMPRED, a software program that makes a prediction of membrane-spanning regions and their orientations; www.ch.EMBnet.org) indicates that the N-terminal hydrophobic domain (residues 3–21) is oriented outside to inside (luminal to cytoplasmic), followed by the CR1-α segment on the cytoplasmic side of the membrane. This model predicts the central transmembrane domain (residues 151–170) to be oriented inside to outside with the CR1-β segment on the luminal side. The C-terminal transmembrane domain (residues 430–455) would thus be oriented outside to inside, followed by a highly basic stop transfer segment. Separately encoded CR1-α and -β proteins likely follow this topology as well.

**Table 2:** E3 region proteins of the 11 macaque adenoviruses belonging to SAdV-B isolates from macaque fecal samples compared with the E3 regions of other macaque adenoviruses*

Adenovirus type	12.5K	CR1-α	CR1-β	RID-α, RID-β, and 14.7K
SAdV-A				
SAdV-3	Present	Present	Present	Present
SAdV-6	Present	Present	Present	Present
SAdV-48	Present	Present	Present	Present
SAdV-B				
A1173	Present	Present	Present	Present
A1285	Present	Present	Present	Present
A1312	Present	Present	Present	Present
A1139	Present	Fused†		Present
A1163	Present	Fused†		Present
A1258	Present	Fused†		Present
A1296	Present	Fused†		Present
A1327	Present	Fused†		Present
A1335	Present	Fused†		Present
SAdV-49	Present	Fused†		Present
SAdV-50	Present	Fused†		Present
HAdV-G				
SAdV-1	Present	Present	Present	Present
HAdV-52	Present	Present	Present	Present
SAdV-7	Present	Not present	Not present	Not present
Not yet classified				
SAdV-18	Present	Not present	Not present	Not present
SAdV-20	Present	Present	Present	Present

*SAdV, simian adenovirus; HAdV, human adenovirus. †Some members of SAdV-B were found to have a novel configuration in which 2 CR1 proteins were fused into a single open reading frame (see Figure 2, Figure 3).

Of the newly sequenced adenoviruses, SAdV-18 exhibited the shortest E3 region. The SAdV-18 genome encodes only one E3 protein, the homologue of the E3 12.5K protein, for which a function has not yet been determined. In this respect, SAdV-18 is similar to SAdV-7 which also has a severely truncated E3 region that encodes only the E3 12.5 K protein (*17*).

### Analysis of the Fiber Genes

**Figure 4:**
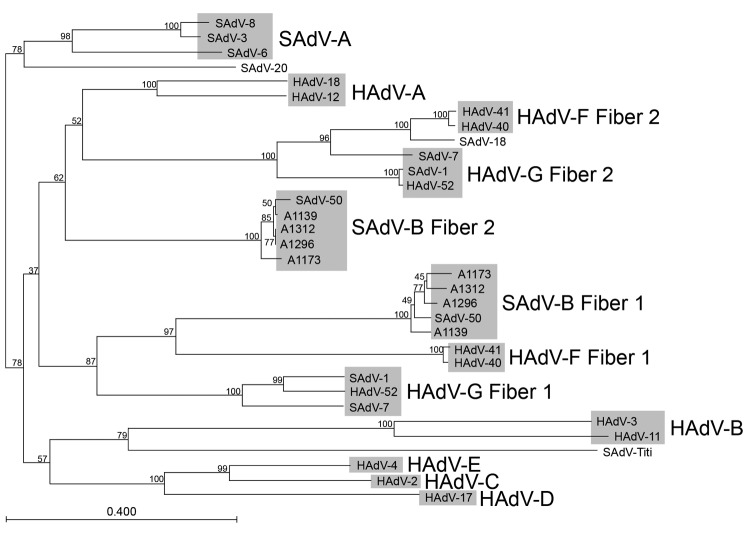
Neighbor-joining alignment of amino acid sequences for the fiber knob domains of macaque adenoviruses (5 representative members of simian adenovirus type B [SAdV-B]) and representative members from each human adenovirus (HAdV) species, with bootstrapping at 1,000 replicates. Alignment was performed by using CLC Bio version 6.1 software (CLC Bio, Aarhus, Denmark). Bootstrap values (percentages) are indicated on the nodes. SAdV-1 and SAdV-7 have been grouped together with HAdV-52 into HAdV-G; the other macaque adenoviruses (except for SAdV-18, SAdV-20, and the titi monkey adenovirus) have been grouped into SAdV-A and SAdV-B. Scale bar indicates number of substitutions per site.

**Figure 5:**
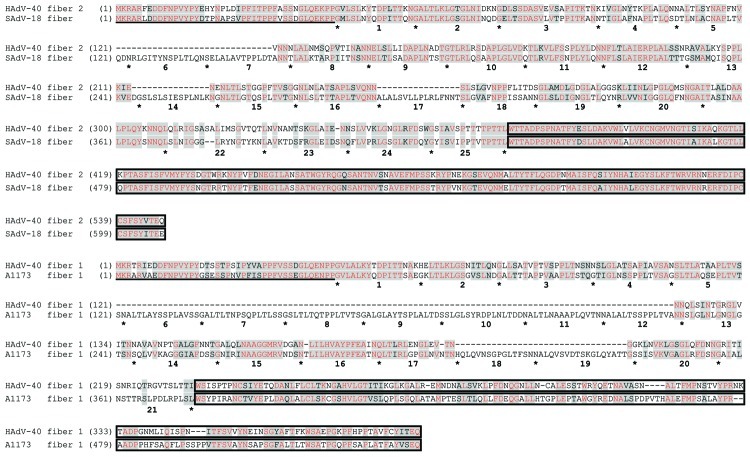
Sequence alignments of the amino acid sequences of human adenovirus (HAdV) 40 long fiber (fiber 2) with simian adenovirus (SAdV) 18 fiber (upper lines) and HAdV-40 short fiber (fiber 1) with macaque adenovirus isolate A1173 (lower lines). Gray shading indicates homologous regions, red font indicates identical residues, underlining indicates N-terminal 30 residues that constitute the tail, and boxes indicate C-terminal knob domains. Intervening shaft domains harboring varying numbers of the ≈16-residue β-spiral repeat sequences are separated by asterisks and numbered sequentially.

The fiber knob domain mediates the initial virus-cell interaction by binding to a cellular receptor. A phylogenetic tree generated on the basis of an alignment of the fiber knob domains of macaque and human adenoviruses is shown in Figure 4. It is evident that the knob domains of the long fiber (fiber 2) of the human adenoviruses belonging to HAdV-F are more similar to those of SAdV-18 than to any other human adenovirus. The SAdV-18 fiber sequence is very similar to the HAdV-F long fiber throughout its length, and the sequence similarity between the knob domains of HAdV-40 and those of SAdV-18 is >90% (Figure 5. HAdV-F species (HAdV-40, HAdV-41, and serologically related isolates) are known to be enteric adenoviruses that frequently cause diarrhea in infants. The HAdV-F long fiber knob can bind the cellular receptor CAR (*22*), and the SAdV-18 fiber knob would probably be able to do so as well. Notably, the shaft domain of SAdV-18 is 391 residues long (the longest such domain of any primate adenovirus sequenced) and harbors as many as 25 iterations of the β-spiral repeat motif (Figure 5). However, unlike HAdV-F, SAdV-18 contains a single fiber gene.

## Discussion

All of the SAdV-B adenoviruses we isolated from macaque fecal samples harbored 2 fiber genes. As discussed above, the only adenoviruses readily isolated from humans that harbor 2 fiber genes belong to HAdV-F (*23**–**25*). The 2 fibers of HAdV-F isolates differ in the lengths of the shaft domain: the first fiber ORF harbors a short shaft (187 residues comprising 12 iterations of the ≈16 amino-acid repeat motif, pfam 00608) and the second fiber gene possesses a longer shaft (330–346 residues, comprised of 21 or 22 iterations of the 16 amino-acid repeat motif) (*23**,**25**,**26*). Both fiber proteins are incorporated into mature virions, with each penton base having either a short or a long fiber protein embedded in them (*23*). Similar to members of HAdV-F, HAdV-G members (macaque adenoviruses SAdV-1 and SAdV-7 and the closely related HAdV-52) also harbor 2 fiber genes. As with HAdV-F, the first fiber gene (fiber 1) encodes the shorter shaft with 9 (SAdV-7) or 10 (SAdV-1 and HAdV-52) motif repeats, and the second fiber gene (fiber 2) encodes the longer fiber shaft, similar in length to that of HAdV-41, which harbors 22 motif repeats. The long fiber of HAdV-F members can bind CAR; a cellular receptor capable of binding the short fiber knob domain has not been identified.

One difference between the newly sequenced adenoviruses we isolated from stool samples (SAdV-B) and previously sequenced adenoviruses with 2 fiber genes (HAdV-40, HAdV-41, SAdV-1, SAdV-7, and HAdV-52) is that first fiber gene (fiber 1) encodes a shaft domain that is longer than the shaft domain of the second fiber gene (fiber 2). The shaft of fiber 1 of the SAdV-B isolates is ≈330 residues long (21 motif repeats, except for SAdV-49 and SAdV-50 which have 1 fewer motif repeat). Except for the length, however, the sequence of the fiber 1 shaft of the SAdV-B isolates is much more similar to the HAdV-F fiber 1 (short) shaft (49.7% sequence identity, 65.6% consensus match; see alignment in Figure 5) than it is to the fiber 1 (short) shaft of SAdV-1 or SAdV-7 (30.8% sequence identity, 47.7% consensus match). The sequence similarity is striking enough to suggest that the HAdV-F short fiber arose by deletions within the shaft domain of a SAdV-B fiber 1–like phylogenetic precursor. In contrast to HAdV-F and HAdV-G members, the second fiber gene of the SAdV-B macaque adenoviruses encodes a shaft domain that is shorter than that of the first fiber, ≈205 residues in length and harboring 14 motif repeats.

For adenoviruses that contain an integrin-binding RGD motif in the penton base proteins, an initial virus-cell interaction mediated by the fiber knob with its receptor (CAR for HAdV-A, C, E and F; CD46 for HAdV-B, and possibly sialic acid for HAdV-D), the penton base–integrin interaction has been shown to be the first step in adenovirus internalization (*27*). Because HAdV-F members do not contain an integrin that binds the RGD motif in their penton base protein, an as-yet-unidentified cellular-binding partner for the short fiber knob domain has been postulated to mediate virus internalization (*27*) in a manner analogous to the penton base–RGD interaction. However, all members of SAdV-A and SAdV-B contain the RGD motif in the penton base protein, which suggests that the short fiber (fiber 1 in HAdV-F and HAdV-G, fiber 2 in SAdV-B) may provide a function that is not necessarily analogous to that provided by the penton base–RGD motif. Moreover, because the knob domain of the SAdV-B fiber 1 gene is “elevated” above the knob on fiber 2, it may play a more critical role in the initial virus-receptor interaction than formerly suspected.

We have previously reported the isolation and sequencing of 3 macaque adenoviruses (SAdV-48, SAdV-49 and SAdV-50) from macaque fecal samples (*9*). Even adenoviral DNA could be detected in most samples by using a sensitive, nested PCR technique, outgrowth of adenoviruses in culture in the monkey cell lines LLC-MK2 or BS-C-1 only ranged from 1% to 16% from various primate colonies. Most of these adenoviruses can be classified into a single subgroup (SAdV-B), although SAdV-A isolates (hexon sequences similar to those of SAdV-48 and SAdV-3) were also identified (Table 1). It is unclear whether these represent a common commensal of the macaque gastrointestinal tract or whether the presence of these adenoviruses in the fecal samples is an artifact of captive status, where virus spread between animals may be more common than in the wild.

The species HAdV-B, HAdV-C, and HAdV-E, which cause acute upper respiratory tract disease in humans, also may set up chronic persistent infections in both humans and apes (*9**,**28*). In contrast, HAdV-F strains, which frequently cause infectious diarrhea in locales with poor sanitation, are only rarely detected in healthy persons (*29*). On the other hand, we have found that monkey adenoviruses belonging to SAdV-A and SAdV-B, as well as SAdV-18 (isolated from primary monkey kidney cells [*2*]), that bear a strong genetic resemblance to HAdV-F do cause chronic infections in otherwise healthy monkeys. HAdV-F may have evolved as a human intestinal pathogen after a recent cross-species transmission event and is thus less well adapted to human hosts than are HAdV-B, HAdV-C, or HAdV-E.

Macaque adenoviruses are usually not thought to infect humans, but the properties of macaque adenoviruses that constitute the species barrier are not known. Recently documented instances of human infections with macaque adenoviruses (*7**,**8*) show that these barriers can sometimes be broken. A more careful investigation of the etiology of infectious diarrhea in areas where monkeys and humans live in proximity (e.g., by PCR of fecal samples followed by sequencing of isolates) could be used to ascertain whether monkey adenoviruses do cause human infections more commonly than is currently surmised.
